# CO_2_-Responsive Graft Modified Chitosan for Heavy Metal (Nickel) Recovery

**DOI:** 10.3390/polym9090394

**Published:** 2017-08-26

**Authors:** Evan A. W. Madill, Omar Garcia-Valdez, Pascale Champagne, Michael F. Cunningham

**Affiliations:** 1 Department of Chemical Engineering, Queen’s University, Kingston, ON K7L 3N6, Canada; evan.madill@gmail.com (E.A.W.M.); omargv86@gmail.com (O.G.-V.); 2 Department of Civil Engineering, Queen’s University, Kingston, ON K7L 3N6, Canada; pascale.champagne@queensu.ca

**Keywords:** nitroxide-mediated polymerization, poly(diethylaminoethyl methacrylate), PDEAEMA, chitosan, grafting, CO_2_-responsive, CO_2_-switchable, wastewater, heavy metal

## Abstract

Chitosan was chemically functionalized with poly(diethylaminoethyl methacrylate) (PDEAEMA) using a grafting to approach to produce a CO_2_-responsive material for adsorbing metals from wastewater streams. A need for improved economical and greener approaches to recover heavy metals from wastewater streams exists due to increasing resource scarcity. Chitosan is currently used as an adsorbent for heavy metals but suffers from some properties that can be disadvantageous to its effectiveness; it is difficult to effectively disperse in water (which limits available surface area) and to regenerate. We set out to improve its effectiveness by grafting CO_2_-responsive tertiary amine containing polymers onto the chitosan backbone, with the goals of preparing and assessing a new type of adsorbent based on a novel concept; using carbon dioxide switchable polymers to enhance the performance of chitosan. PDEAEMA chains prepared by nitroxide-mediated polymerization were grafted onto chitosan functionalized with glycidyl methacrylate. In carbonated water, the grafted chitosan displayed improved dispersibility and exhibited a Ni(II) adsorption capacity higher than several other chemically functionalized chitosan variants reported in the literature with the regenerated material having a higher capacity than all physical and chemical derivatives reported in the literature. The results of this study validate the continued development of this material for applications in heavy metal removal and recovery from wastewater streams.

## 1. Introduction

Chitin (poly(2-acetamido-2-deoxy-β-d-glucose)) is the second most abundant natural polymer in the world, second only to cellulose. This material is produced by a vast number of living organisms, typically as a main component of arthropod exoskeletons [1]. Crab, lobster, and shrimp shells from the food canning industry are currently the dominant feedstock for the production of chitin. Chitosan (CTS) is produced through partial or complete *N*-deacetylation of chitin. Many countries across the world have large unexploited sources of crustacean shells that are appropriate for manufacturing chitin and chitosan. Both biopolymers exhibit excellent biocompatibility, biodegradation in the human body, and non-toxicity making them attractive over synthetic alternatives [2]. These properties allow chitosan to find applications in the water and wastewater treatment, agriculture, biopharmaceuticals, cosmetics and toiletries, and the food and beverages industries [1]. Compared to cellulose and chitin, the ability to dissolve chitosan under aqueous acidic conditions allows for somewhat easier functionalization; however, chitosan does still have only limited solubility in most solvents which poses a major obstacle to more widespread application in various fields including wastewater treatment, cosmetic, and medicinal applications.

Nickel is used in the production of over 3000 different metal alloys. Nickel contamination in water sources arises from both natural and anthropogenic sources, including acid rain and effluents/leachates from electroplating, nickel mining, and smelting operations that can exceed regulatory limits [3,4,5]. Treatment strategies have been proposed. Currently, heavy metals are predominantly precipitated out of wastewaters with the addition of caustic lime [6]. However, this approach essentially relocates the issue into a solid phase, offers limited removal efficiencies, requires additional downstream treatment and is a non-regenerative method that does not recover nickel from the waste stream [3,7,8]. Alternatives to conventional treatment that allow for nickel recovery include membrane separation processes (e.g., reverse osmosis, micro-, ultra- and nanofiltration) and electrochemical processes (e.g., electrodialysis, electrolysis, and electrocoagulation) [3,9,10]. Unfortunately, these treatment processes utilize expensive equipment that require continual maintenance and are energy intensive [5,10]. Several natural adsorbents have been reported in the literature that offer a simpler, low cost, and easily operated solution for nickel removal [10]. Some of these materials can be regenerated for reuse using caustic or acid agents [11]; however, they can be plagued by variability in their efficacy.

Chitosan is a well-known adsorbent of heavy metals [1,2,12,13,14]. Metal adsorption onto chitosan is assumed to occur through either chelation or coordination with amino groups or ion exchange with protonated amino groups. Both physical modification of chitosan (e.g., formation of gels, coating PVC beads, and membranes) and chemical functionalization (e.g., crosslinking, and grafting with chelating agents, crown ethers, and synthetic polymers) have been reported [1,2,13,15]. Chemical functionalization of chitosan is dominated by grafting at the amino functionality of the chitosan rather than the hydroxyl functionality because of the higher reactivity of the amino group, but the amino group is then unavailable for subsequent use, which may not be desirable [2,15]. The graft-modification of chitosan and other natural polymers using living radical polymerization techniques (Reversible Deactivation Radical Polymerization, RDRP) has recently been reviewed [16,17].

In this investigation chitosan was functionalized with poly((diethylamino)ethyl methacrylate (PDEAEMA) via nitroxide-mediated polymerization (NMP) yielding chitosan-grafted PDEAMEA (CTS–*g*–GMA–PDEAMEA), to improve its effectiveness by grafting CO_2_-responsive tertiary amine containing polymers onto the chitosan backbone, with the goals of preparing and assessing a new type of adsorbent based on a novel concept; using carbon dioxide switchable polymers to enhance the performance of chitosan, The longer term objective is development of a material that allows for the recovery of the adsorbed metal and regeneration of the material without the use of aggressive acid or caustic agents. Recently, our research group published new techniques for grafting a range of synthetic polymers both to and from the hydroxyl group of chitosan using NMP [18,19] and also examined the use of graft-modified chitosan for dye removal from aqueous streams [20]. Polymers containing tertiary amines have been shown to have heavy metal adsorption capabilities and have been a focus of our research group for their ability to “switch” their hydrophilicity through gassing and degassing of carbon dioxide [21,22]. PDEAEMA was selected as the synthetic polymer to be grafted *to* chitosan because of its water-solubility in carbonated water and its CO_2-_switchability which allows it to be easily and reversibly converted from a water-soluble to water-insoluble polymer simply by removing CO_2_ (i.e., by bubbling air or nitrogen) [23]. Compared to the grafting to route developed in our laboratory that focused on water-insoluble polymers [18], the use of a water-soluble polymer in this work eliminated the need to first functionalize the chitosan with SDBS (sodium dodecyl benzene sulfonate), thereby significantly reducing the use of organic solvents.

It was hypothesized that, similar to chitosan, the mechanism of metal adsorption by the tertiary amines would occur via chelation. Conversely, CO_2_-induced switching of the grafted chitosan (i.e., protonation of the tertiary amines) with CO_2_ would release the metal, allowing for its recovery. Regeneration of the grafted chitosan via gassing with air or nitrogen would then return the CTS–*g*–GMA–PDEAMEA adsorbent to its initial state, ready for reuse. This recovery and regeneration process could offer potential environmental and economic advantages over the use of acid and caustic agents. Nickel was chosen as the target heavy metal not only because of its wide use, but also as a representative of other metals (i.e., arsenic, cadmium, cobalt, chromium, lead, nickel, and lead), which could also possibly be treated using this method. Beyond the scope of this work, this material could potentially offer an inexpensive and greener treatment and recovery approach for heavy metals that is readily tunable to different wastewater conditions through use of selected synthetic polymers.

## 2. Materials and Methods

### 2.1. Materials

Chitosan (CTS, Aldrich, degree of deacetylation of 85%, St. Louis, MO, USA), glycidyl methacrylate (GMA, Aldrich, 97%), hydroquinone (Fisher, Hampton, NH, USA), acetic acid (Fisher, 99.7%), tetrahydrofuran (THF, ACP, 99+%, Philadelphia, PA, USA), and deuterium oxide (Cambridge Isotope Laboratories, D 99.9%, Tewksbury, MA, USA) were used as received. Styrene (St, Aldrich, 99+%) and 2-(diethylamino)ethyl methacrylate (DEAEMA, Aldrich, 99%) were passed over a column containing basic aluminum oxide (Aldrich, ~150 mesh, 58 Å) to remove the inhibitor and stored below 5 °C prior to polymerization. *N*,*N*′-dicyclohexylurea was purchased from Aldrich and used as received. SG1 (*N*-tert-butyl-*N*-(1-diethylphosphono-2,2-dimethylpropyl) nitroxide) (85%, was kindly supplied by Arkema, Colombes, France). *N*-Hydroxysuccinimide-BlocBuilder (NHS-BB) was synthesized from BlocBuilder (BB, *N*-(2-methylpropyl)-*N*-(1-diethylphosphono-2,2-imethylpropyl)-*O*-(2-carboxylprop-2-yl) hydroxylamine (BB, 99%, provided by Arkema) according to a previously reported procedure [24], as follows: BB (5 g, 13.1 mmol) and *N*-hydroxysuccinimide (1.81 g, 15.7 mmol) were dissolved in THF (20 mL) and deoxygenated by bubbling nitrogen for 15 min. Then, a degassed solution of *N*,*N*′-dicyclohexylcarbodiimide (3 g, 14.4 mmol) in THF (5 mL) was added. After stirring at 0 °C for 1.5 h, the precipitated *N*,*N*′-dicyclohexylurea was removed by filtration and the filtrate volume was reduced under vacuum to one third and placed at −20 °C for 2 h in order to precipitate the residual *N*,*N*′-dicyclohexylurea [25]. After filtration, the solution was concentrated under reduced pressure and precipitation was performed in pentane. The obtained solid was further washed with water to remove *N*-hydroxysuccinimide. After drying under vacuum, alkoxyamine 1 was obtained as a white powder. Nickel sulphate (NiSO_4_·6H_2_O, Aldrich, 99%), concentrated nitric acid (Aldrich, 68.0–70%) and carbon dioxide gas (MEGS, bone dry 99.8%) were employed for the equilibrium adsorption studies.

### 2.2. Instrumentation

^1^H NMR spectroscopy was performed on an FT-NMR Bruker Avance 400 MHz spectrometer (Billerica, MA, USA), with a total of 256 scans, at room temperature using D_2_O/CH_3_COOH 0.4 M as a solvent at 5 mg/mL. Thermogravimetric analysis (TGA) was performed using a TA Instruments Q500 TGA analyser (New Castle, DE, USA) by heating the sample using the following temperature ramp: 10 °C min^−1^ from 30 °C to 75 °C, held for 30 min at a plateau of 75 °C, and 10 °C min^−1^ to 600 °C. Gel Permeation Chromatography (GPC) analysis was performed with a Waters 2690 Separation Module and Waters 410 differential refractometer with THF as the eluent (Aldrich, Oakville, ON, Canada). The column bank consisted of Waters Styragel HR (4.6 mm × 300 mm) 4, 3, 1, and 0.5 separation columns at 40 °C. pH measurements were completed with a Thermo Fisher Scientific Inc. Orion Star A211 Benchtop Meter (Waltham, MA, USA). Agitation for equilibrium adsorption studies was completed with a 120 V, VWR International LLC (Suwanee, GA, USA). Standard Orbital shaker model 5000 (shaker table, run at 150 rpm).

### 2.3. Synthesis of Chitosan–g–glycidyl Methacrylate (CTS–g–GMA)

**Scheme 1:**
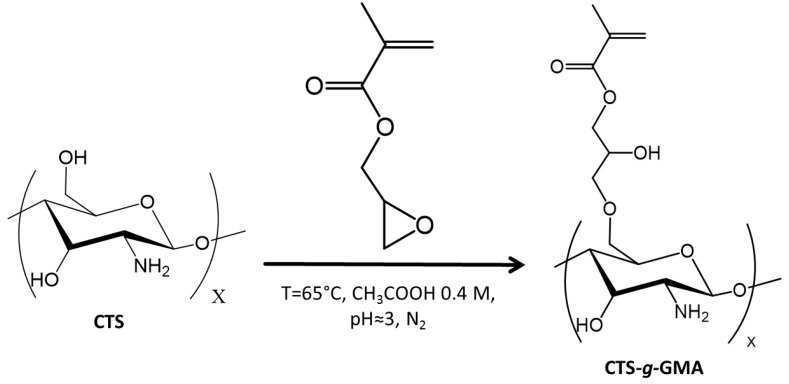
Functionalization of chitosan with glycidyl methacrylate (GMA).

CTS–*g*–GMA was synthesized using a methodology developed by Garcia-Valdez et al. [19] (Scheme 1). Chitosan (1.0 g) was dissolved in 100 mL 0.4 M acetic acid solution (2.28 mL in 100 mL of de-ionized water) in a 250 mL three neck round bottom flask. After the additions of 5 mL of 0.05 M KOH (0.014 g in 5 mL of deionized water) solution and 10 mL of 9.08 mM hydroquinone (0.01 g in 10 mL of deionized water) solution, the mixture was degassed under nitrogen for 30 min then heated to 65 °C. GMA (24.0 mmol, 3.30 mL) was injected into the flask dropwise and the mixture was magnetically stirred for 2 h under these conditions. The solvent was removed under vacuum and the product was washed twice with THF (~80 mL), once with de-ionized water (~80 mL) and filtered. CTS–*g*–GMA was analyzed by ^1^H NMR (degree of functionalization of 11 mol %).

### 2.4. Synthesis of Poly(diethylamino)ethyl Methacrylate (PDEAEMA) via Nitroxide Mediated Polymerization (NMP)

**Scheme 2:**
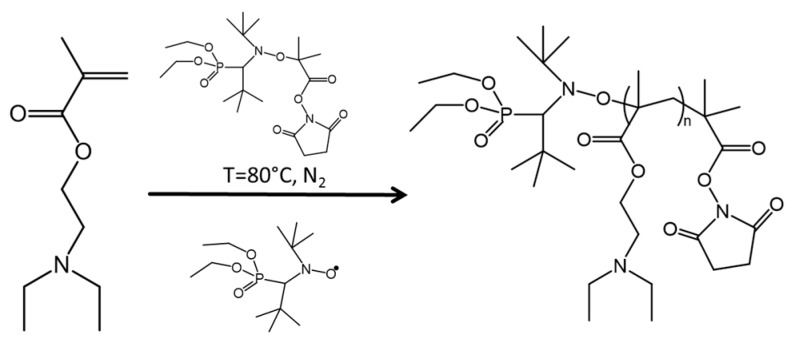
Synthesis of poly(diethylaminoethyl methacrylate) (PDEAEMA) via nitroxide-mediated polymerization (NMP).

Polymerization of DEAEMA was completed using NMP (Scheme 2). In a 250 mL three neck round bottom flask, the uninhibited DEAEMA (0.26 mol, 47.57 mL), styrene (0.03 mol, 3.47 mL), NHS-BlocBuilder^®^ (2.9 mmol, 1.4038 g) and SG1 (0.32 mmol, 0.1022 mL) were magnetically stirred and degassed under nitrogen for 30 min. The mixture was stirred at 80 °C for 1 h, with 2 mL samples extracted every 20 min for GPC analysis. PDEAEMA was precipitated in hexanes cooled with liquid nitrogen, decanted, washed in THF (~80 mL), re-precipitated in hexanes and decanted. The product was analyzed by GPC and TGA (yield of 53% monomer conversion).

### 2.5. Synthesis of CTS-g-GMA-PDEAEMA

**Scheme 3:**
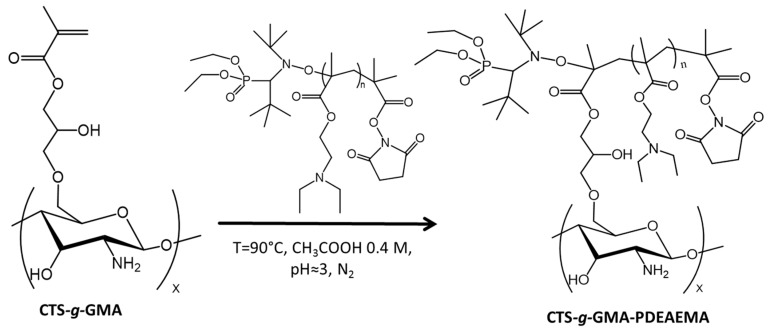
Grafting PDEAEMA to chitosan-grafted PDEAMEA (CTS–*g*–GMA–PDEAMEA).

Grafting to synthesis (Scheme 3) was conducted using the following procedure. In a 250 mL three neck round bottom flask, CTS–*g*–GMA (1.218 g) was dissolved in 100 mL of 0.1 M acetic acid (0.57 mL in 100 mL of de-ionized water) solution. The pH of the solution was then adjusted to 5.0 using 1.0 M KOH (0.280 g in 50 mL of deionized water) solution, degassed for 30 min under a nitrogen atmosphere and heated to 90 °C. The polymer (2.40 g) was dissolved in 60 mL of 0.1 M acetic acid (0.340 mL in 100 mL of deionized water) solution. The polymer solution was added in semibatch increments of 20 mL to the heated flask and magnetically stirred for 1 h before the next addition. After cooling, the solvent was evaporated under vacuum. The product was washed twice with THF (~80 mL), once with de-ionized water (~80 mL) and vacuum dried. Products were analyzed using TGA and ^1^H NMR. From ^1^H NMR analysis it was determined a reaction yield of 2.7 chains of PDEAEMA per 100 units of CTS.

### 2.6. Ni(II) Adsorption Equilibrium Studies

Equilibrium adsorption studies were conducted using a combination of previously reported procedures [26,27]. One litre stock solutions of 50, 200, 500. and 1000 mg/L Ni(II) were prepared by adding the appropriate weight of NiSO_4_·6H_2_O to deionized water to obtain Ni(II) concentrations of 50, 200, 500, and 1000 mg/L. The solution was stirred for 15 min with a magnetic star bar (size: 2.5 cm × 0.8 cm) at 350 rpm to achieve complete dissolution at room temperature conditions.

For simple adsorption isotherm trials, an initial 5 mL sample was collected and acidified with two drops of nitric acid to preserve the sample for subsequent metal analysis. Next, 50 mg of absorbent (grafted chitosan) and 100 mL of the desired concentrated solution were added to a 250 mL beaker. After recording the initial pH, the beakers were covered and agitated at 150 rpm on an orbital shaker table for a contact period of 24 h. The final pH of the samples was recorded and 10 mL samples were collected, filtered using 0.2 µm PTFE syringe filters and acidified with two drops of concentrated nitric acid to preserve the sample. The equilibrium adsorption studies were conducted in triplicate.

For trials that involved pH adjustment via CO_2_ gassing, after an initial pH reading, 1 h of CO_2_ gassing was completed and the final pH recorded. An initial 5 mL sample was collected and acidified with two drops of nitric acid to preserve the sample for subsequent metal analysis. Adsorption was performed as mention in the previous paragraph. Metal concentrations were determined via ICP-OES analysis. For each adsorbent, the unit equilibrium adsorption capacity ($q_{e}$, mg/g) was calculated using Equation (1).

(1) $$q_{e}=\left(\frac{C_{o}-C_{e}}{m}\right)\cdot V$$

where $C_{o}$ and $C_{e}$ were the initial and equilibrium (final) metal concentrations (mg/L), m was the mass of adsorbent (g), and V was the volume of solution (L).

The experimental data was fit to the Langmuir Equation (2) and Freundlich isotherms Equation (3).

(2) $$q_{e}=\frac{q_{max}C_{e}K_{L}}{1+C_{e}K_{L}}$$

(3) $$q_{e}=K_{F}C_{e}^{1/n}$$

where $K_{L}$ is the Langmuir isotherm constant (L/g), $q_{max}$ is the theoretical maximum adsorbance (mg/g), $K_{F}$ is the Freundlich isotherm constant (L/g) and 1/n is the dimensionless Freundlich isotherm exponential constant.

### 2.7. CO_2_ Regeneration Studies

After completing the initial equilibrium adsorption studies, the remaining solution containing the grafted chitosan was filtered using Whatman 1 filter paper and washed with deionized water (~20 mL). The grafted chitosan was collected and placed in an Erlenmeyer flask containing 20.0 mL of deionized water. Initial pH was recorded, the flasks were covered and CO_2_ gas was bubbled into the solution for 1 h of gassing. The final pH was recorded, a 10 mL sample was collected, filtered using 0.2 µm PTFE syringe filters and acidified with two drops of concentrated nitric acid to preserve the sample for subsequent metal analysis. Metal concentrations were determined via ICP-OES analysis. The remaining solution was filtered using Whatman 1 filter paper and the grafted chitosan was collected and allowed to air-dry overnight.

## 3. Results & Discussion

### 3.1. Characterization of CTS–g–GMA

**Figure 1:**
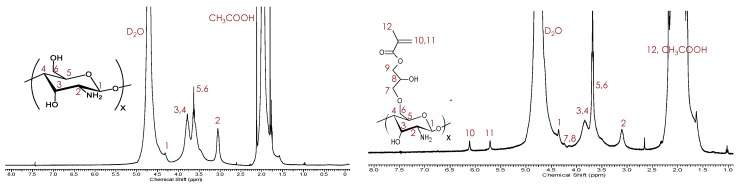
^1^H NMR collected in 0.4 M CH_3_COOH/D_2_O (5 mg/mL) of pure chitosan (**left**) and CTS–*g*–GMA (**right**).

CTS–*g*–GMA was synthesized by reacting the GMA epoxide with the primary alcohol from chitosan. Protection of the amino groups was achieved by performing the reaction under acidic conditions (pH = 3.8) such that the amino groups were protonated and therefore protected. Success of the reaction was verified by ^1^H NMR analysis after removal of the residual monomer from solution by exhaustive washing. Peak assignments for pure chitosan (Figure 1, left) versus CTS–*g*–GMA (Figure 1, right) revealed the presence of the GMA vinyl protons (H_10_—6.1 ppm and H_11_—5.7 ppm) in the grafted product. The degree of functionalization of chitosan with GMA was estimated to be 11 mol %, based on the integral ratio between the GMA vinyl proton peak (H_10_—6.1 ppm), and the lone proton alpha to the primary amine (H_2_—3.1 ppm) [18,19,20].

### 3.2. Characterization of PDEAEMA

**Figure 2:**
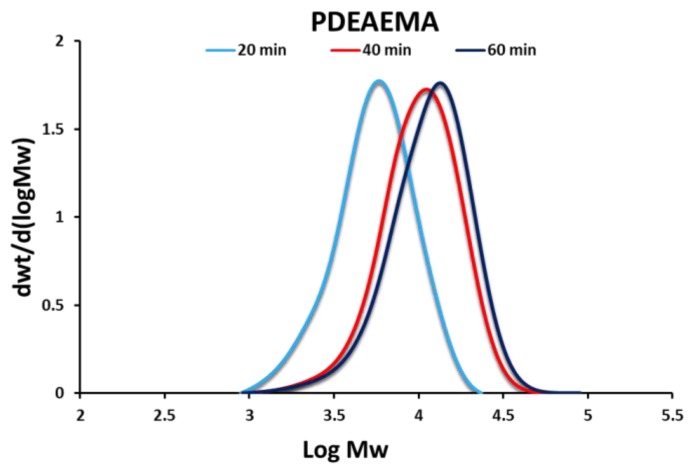
Gel Permeation Chromatography (GPC) traces of SG1-terminated PDEAEMA at various reaction times. DEAEMA (0.26 mol, 47.57 mL), styrene (0.03 mol, 3.47 mL), NHS-BlocBuilder^®^ (2.9 mmol, 1.4038 g), SG1 (0.32 mmol, 0.1022 mL). Polymerization was run at 80 °C for 1 h.

PDEAEMA was synthesized via NMP. A 53% conversion of monomer into polymer was achieved, as determined gravimetrically. The narrow molecular weight distribution (MWD) and evolution of the MWD (Figure 2) indicated a well-controlled living polymerization (the majority of the polymer chains were terminated with SG1, which is required to initiate the grafting to reaction in the subsequent step). The number average molecular weight (*M*_n_) was 9710 g/mol, with a dispersity (*D*) of 1.37.

### 3.3. Characterization of CTS–g–GMA–PDEAEMA

**Figure 3:**
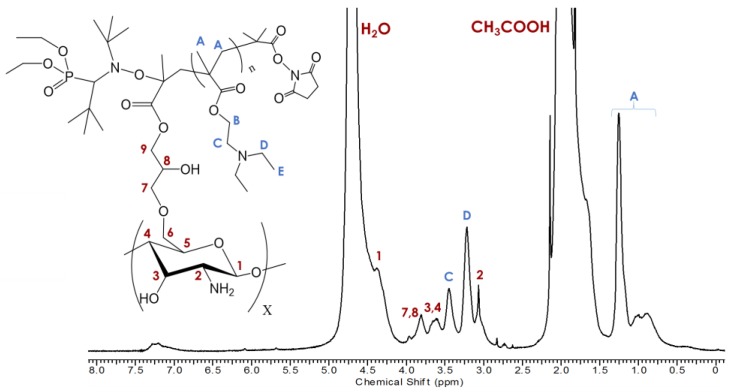
^1^H NMR collected in 0.4 M CH_3_COOH/D_2_O at 5 mg/mL of CTS–*g*–GMA–PDEAEMA with peak assignments.

Thermal dissociation of the SG1 from the PDEAEMA chain results in the formation of two radicals: the stable SG1-nitroxide radical and a free radical at the end of the polymer chain. This chain-end radical can react with the vinyl group of CTS–*g*–GMA before being reversibly deactivated by recombination with SG1, covalently linking the polymer chain to the chitosan. Success of the reaction was validated by ^1^H NMR and TGA after removal of residual DEAEMA monomer and free polymer chains from solution by exhaustive washing. Characteristic signals belonging to PDEAEMA including the methacrylate methyl protons (H_A_—1.25 ppm) and the two nitrogen shifted ethylene protons (H_C_—3.45 ppm and H_D_—3.13 ppm) are evident in the ^1^H NMR (Figure 3). Signals from the GMA vinyl groups were still present at 7.2–7.3 ppm indicating that not all GMA groups were grafted with PDEAEMA [18,19,20].

**Figure 4:**
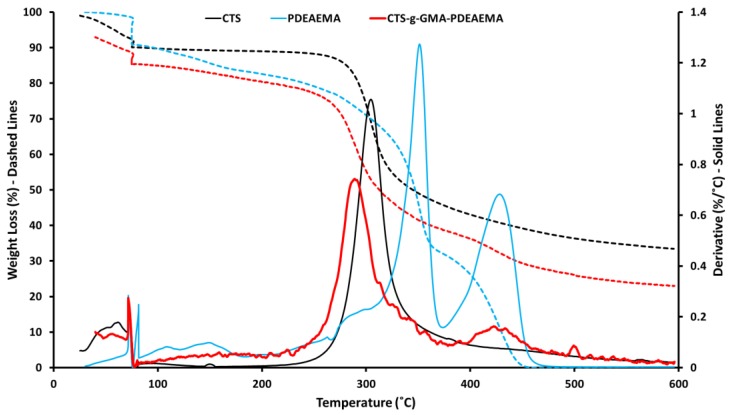
Thermogravimetric analysis (TGA) traces for pure and grafted chitosan.

TGA analysis (Figure 4) indicated that decomposition of pure chitosan occurred at 250–350 °C, while decomposition of PDEAEMA occurred in two steps at 320–370 °C, attributed to the decomposition of functional group (diethylaminoethyl) and 390–450 °C attributed to the decomposition of the main backbone chain. CTS–*g*–GMA–PDEAEMA was found to exhibit two decomposition peaks. The first peak was observed at 250–370 °C and corresponds to the chitosan backbone chain with the broadening of the signal a result of the PDEAEMA peak from 320–370 °C. The second peak noted at 390–450 °C was attributed to presence of grafted PDEAEMA, as this signal was not present for the pure chitosan sample analysis. CTS, PDEAEMA, and CTS–*g*–GMA–PDEAEMA showed an initial weight loss in the range of 70–100 °C which is attributed to adsorbed water. The combination of characteristic ^1^H NMR and TGA signals confirmed that PDEAEMA had been successfully grafted to the chitosan.

### 3.4. CO_2_ Regeneration Study

To determine the response of CTS–*g*–GMA–PDEAEMA to CO_2_, 0.10 g of the material was placed in 15 mL of deionized water and sparged with CO_2_ for 1 h to protonate the CTS–*g*–GMA–PDEAMEA adsorbent. Next, air was bubbled through the mixture for 1 h to deprotonate and regenerate the CTS–*g*–GMA–PDEAMEA adsorbent. For comparison, the same procedure was applied using pure chitosan.

**Figure 5:**
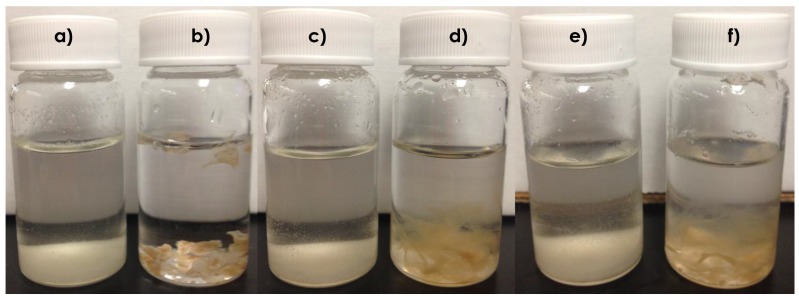
CO_2_ switchability tests: (**a**) chitosan in water (pH = 8.74); (**b**) CTS–*g*–GMA–PDEAMEA in water (pH = 7.18; (**c**) chitosan after 1 h of CO_2_ bubbling (pH = 4.96); (**d**) CTS–*g*–GMA–PDEAEMA after 1 h of CO_2_ bubbling (pH = 5.42); (**e**) chitosan (pH = 8.12) after 1 h of CO_2_ bubbling (pH = 5.42), followed by 1 h of air bubbling; (**f**) CTS–*g*–GMA–PDEAMEA (pH = 7.32) after 1 h of CO_2_ bubbling (pH = 5.42), followed by 1 h of air bubbling.

As can been seen from Figure 5b, prior to CO_2_ sparging, the CTS–*g*–GMA–PDEAMEA adsorbent appeared as a solid precipitate in the water. After CO_2_ was bubbled through the system, the material was observed to swell, become more translucent and had a gelatin-like consistency (Figure 5d). Bubbling air through this swollen system increased the pH, and presumably deprotonated at least some of the CO_2_, however the material remained swollen (Figure 5f), even though the pH returned to its original value. Visually these results indicate that CTS–*g*–GMA–PDEAMEA is CO_2_ responsive, with the grafted PDEAEMA protonated by carbonic acid upon CO_2_ sparging. It is noteworthy that after being protonated once, the grafted chitosan retains its highly swollen state, suggesting the effective surface area of the chitosan and PDEAEMA available for metal complexation should be notably higher than for the unswollen chitosan shown in Figure 5b. The results of the adsorption studies (discussed in the following section) corroborate this observation. The similarity in appearance of the chitosan samples (Figure 5a,c,e) indicated that chitosan alone was not CO_2_ responsive.

### 3.5. Ni(II) Adsorption Equilibrium Studies

**Figure 6:**
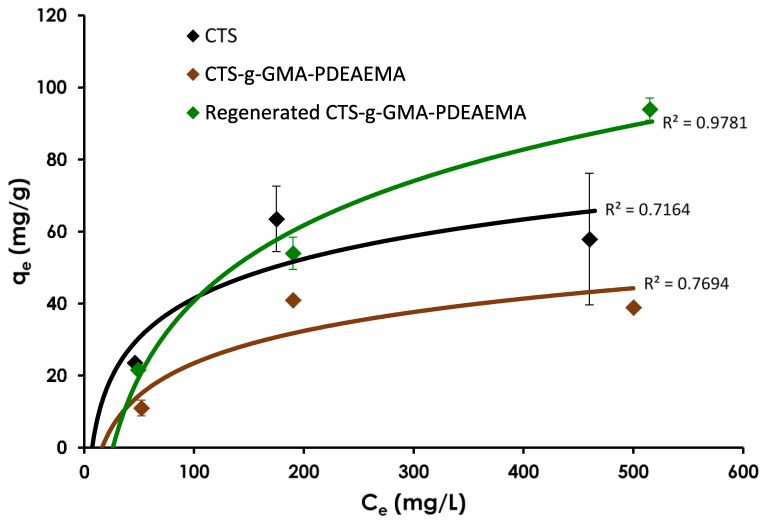
Equilibrium Ni(II) adsorption capacity for chitosan, pure and regenerated CTS–*g*–GMA–PDEAEMA as a function of equilibrium concentration. Initial pH of adsorption trials was 5.80–5.87 and final was 7.40–7.81. Addition of spent CTS–*g*–GMA–PDEAEMA to de-ionized water prior to CO_2_ sparging yielded a pH of 8.03–8.79. After 1 h of CO_2_ sparging the pH decreased to 5.35–5.68.

The first Ni(II) adsorption equilibrium study compared the adsorption capacity of chitosan to CTS–*g*–GMA–PDEAEMA without pH adjustment (i.e., tertiary amine groups were not protonated). It is important to mention that it has been established in the literature that pH plays an important role in the adsorption capacities of different materials [28,29,30,31], however in our particular system, due to the nature of the CO_2_ (gas) it was not possible to control the pH. These trials (Figure 6) suggested that the initial CTS–*g*–GMA–PDEAEMA was not as strong an adsorbent as pure chitosan despite the addition of 1.35 PDEAEMA chelation sites for every one chitosan chelation site. This was likely due to the steric bulk of the hydrophobic PDEAEMA polymer chains attached to the chitosan backbone reducing the accessibility to all possible chelation sites. Regeneration of the material with 1 h of CO_2_ sparging led to the release of 61% (average) of the nickel that had been previously adsorbed on the CTS–*g*–GMA–PDEAEMA. After regeneration with CO_2_, the adsorption capacity of the grafted material increased by 196%–241%, surpassing pure chitosan at nickel equilibrium concentrations beyond ~100 mg/L. This could be attributed to the protonation of the amine groups on the PDEAMA chains that would allow for better swelling and dispersion of the CTS–*g*–GMA–PDEAEMA, thereby facilitating interactions between the nickel ions and the chelation sites. The increase in adsorption capacity after regeneration also suggested that the material was not completely protonated such that all or most of the PDEAEMA chelation sites were unavailable for chelation. The increased adsorption was the motivation for the subsequent trials where the adsorbent materials were initially sparged with CO_2_ as a “conditioning” step prior to measuring adsorption capacity.

In the results, some variability was anticipated due to the inherent natural nature of the chitosan, which was evident from the relative standard errors computed for chitosan (3.2–40.7%) compared to the CTS–*g*–GMA–PDEAEMA (0.4–19.9%). This would suggest that the addition of a synthetic polymer (PDEAMA) reduces the variability in its removal efficiency.

During the chitosan adsorption trials, the unmodified chitosan became very finely dispersed under acidic conditions. As such, the recovery of unmodified chitosan would require a finer filter and higher pressures compared to the coarse filter used for CTS–*g*–GMA–PDEAEMA that was easily filtered under gravity. Hence, in terms of industrial or municipal applications, the recovery of CTS–*g*–GMA–PDEAEMA as a regenerated adsorbent would likely be less difficult and more economical compared to the unmodified chitosan.

### 3.6. Ni(II) Adsorption Modelling

The equilibrium data was fit to two of the simplest adsorption isotherm models: the Langmuir and Freundlich models. Monolayer adsorption is assumed in the Langmuir model compared to the empirical Freundlich model that is based on adsorption to a heterogeneous surface. Both of these models have been applied to chitosan and its derivatives in other studies [27,32,33]. The adsorption isotherm parameters derived from the experimental data in this study are summarized in Table 1.

Published literature values for reported equilibrium adsorption capacities of Ni(II) onto chitosan and its derivatives are presented in Table 2. The adsorption capacity of chitosan has been noted to be a function of pH [27]. The calculated maximum adsorption capacity we obtained for CTS–*g*–GMA–PDEAEMA prior to CO_2_ conditioning was within the range of cited values for pure chitosan. However, after being regenerated (i.e., after CO_2_ conditioning), the CTS–*g*–GMA–PDEAEMA prepared in this study exhibited a much higher adsorption capacity than several of the other chemically functionalized chitosan variants reported in the literature.

## 4. Conclusions

Chitosan was successfully chemically functionalized with CO_2_-responsive PDEAEMA. The grafted material exhibited CO_2_ switchability, that chitosan alone did not. The equilibrium adsorption capacity of CTS–*g*–GMA–PDEAEMA was initially (prior to conditioning with CO_2_) found to be slightly lower than that of chitosan, but after regeneration showed the highest adsorption capacity of all trials. Furthermore, the regenerated material exhibited the highest adsorption capacity of all comparable materials published in the literature. The average nickel release of 61% from the CTS–*g*–GMA–PDEAEMA was comparable to established methods using chitosan with acid and caustic agents or EDTA as regenerating agents, but required only CO_2_ sparging. In addition, further development offers promise for the use of CO_2_-responsive grafted chitosan as an economical and greener approach for the recovery of dissolved metals, in this case Ni(II), from aqueous wastewater streams.

## Figures and Tables

**Table 1:** Langmuir and Freundlich isotherm (20 °C) parameters derived for the adsorption of Ni(II) onto chitosan and CTS–*g*–GMA–PDEAEMA for an initial concentration range of 50–1000 mg Ni(II)/L, with SO_4_^2−^ as the anion.

Adsorbent	pH	Langmuir Isotherm		Freundlich Isotherm	
		Theoretical $q_{max}$ (mg/g)	$K_{L}$ (L/g)	$K_{F}$ (L/g)	1/n
CTS	5.80–5.87	66.2	1.91 × 10^−2^	5.55	0.744
CTS-*g*-GMA-PDEAEMA	5.80–5.87	51.3	7.57 × 10^−3^	1.29	0.112
CTS-*g*-GMA-PDEAEMA with initial CO_2_ sparging alone	4.56–4.83	59.9	1.52 × 10^−2^	5.45	0.736
Regenerated CTS-*g*-GMA-PDEAEMA	5.80–5.87	149.3	3.25 × 10^−3^	1.92	0.283

**Table 2:** Equilibrium adsorption capacities of Ni(II) for chitosan, physical and chemically functionalized chitosan.

Adsorbent	Equilibrium Adsorption Capacity (*q*_e_, mg/g)	pH
CTS [34]	2.4	5.0
CTS coated PVC beads [27]	120.5	5.0
CTS coated perlite [35]	114.9	5.0
CTS–*g*–EDTA [36]	123.3	2.0
CTS–*g*–Acetate crown ether [37]	0.7	5.6
CTS–*g*–Diacetate crown ether [37]	4.1	5.6
Epichlorohydrine cross-linked CTS [37]	6.4	5.6
Epichlorohydrine cross-linked CTS–*g*–Histidine [38]	140.8	5.0
